# Psychosocial and Organizational Determinants of Perceived Occupational Exposure and Job Satisfaction Among Intensive Care Unit Nurses

**DOI:** 10.3390/healthcare14162614

**Published:** 2026-08-19

**Authors:** Katarzyna Tomaszewska, Bożena Majchrowicz, Helena Kadučáková, Marcela Ižová, Piotr Kołodziej, Krystyna Kowalczuk

**Affiliations:** 1 Department of Nursing, Institute of Health Protection, The Bronisław Markiewicz Academy of Applied Sciences, 37-500 Jarosław, Poland; 2 Institute of Nursing, Faculty of Health Sciences and Psychology, Collegium Medicum, University of Rzeszów, 35-959 Rzeszów, Poland; 3 University Research and Development Center in Health Sciences, Faculty of Health Sciences and Psychology, Collegium Medicum, University of Rzeszów, 35-959 Rzeszów, Poland; 4 Department of Nursing, Faculty of Health Sciences, Catholic University in Ružomberok, 034 01 Ružomberok, Slovakia; 5 Department of Health Care Sciences, Faculty of Humanities, Tomas Bata University in Zlin, 760 01 Zlín, Czech Republic; 6 Department of General Psychiatry, Fryderyk Chopin University Clinical Hospital in Rzeszów, 35-055 Rzeszów, Poland; 7 Department of Integrated Medical Care, Medical University of Białystok, 15-089 Białystok, Poland

**Keywords:** work environment, psychosocial factors, ICU nurses, occupational exposure, job satisfaction

## Abstract

**Introduction:** Intensive care units are characterized by a high level of clinical and psychosocial demands. Previous research indicates that the work environment plays a significant role in shaping nurses’ occupational well-being; however, analyses that simultaneously consider organizational factors, perceived occupational exposure, and job satisfaction are still limited, particularly in the context of Central and Eastern European countries. **Objectives:** The aim of the study was to assess the relationships between psychosocial and organizational factors in the work environment and the level of perceived occupational exposure as well as job satisfaction among nursing staff working in intensive care units. **Methods:** The study was cross-sectional in design and was conducted in Poland among 226 nurses employed in intensive care units. Data were collected using an anonymous online survey that included an author-designed questionnaire and the standardized “Working Conditions” instrument developed by the Central Institute for Labor Protection—National Research Institute. Data analysis was performed using descriptive statistics, chi-square (χ^2^) tests, and binary logistic regression. The study was conducted and reported in accordance with the STROBE guidelines (Strengthening the Reporting of Observational Studies in Epidemiology). **Results:** A high psychosocial burden was observed in 49.6% of the participants, while a high organizational risk was found in 69.9%. Higher organizational risk was associated with a more than threefold increase in the odds of high perceived occupational exposure (OR = 3.47; 95% CI: 1.54–7.81). **Conclusions:** Psychosocial and organizational factors in the work environment play an important role in shaping perceived occupational exposure and job satisfaction among intensive care nursing staff. The results highlight the importance of organizational interventions, such as ensuring adequate nurse staffing, strengthening supportive leadership, and developing psychosocial support programs for staff.

## 1. Introduction

Intensive care units (ICUs) constitute high-acuity care environments with substantial clinical and organizational demands, in which nursing staff operate under conditions of considerable variability and unpredictability in patients’ status and persistent time pressure. ICU work requires rapid clinical decision-making for patients with life-threatening conditions, a high level of responsibility for patient safety, and continuous readiness to respond to emergencies. These factors place ICU nurses among occupational groups particularly exposed to psychosocial strain stemming from the nature of critical care, shift work, and frequent exposure to end-of-life and other boundary situations [1].

International studies indicate that symptoms of burnout affect up to 30–50% of intensive care unit nurses, while more than half of ICU staff report experiencing verbal or physical violence, confirming the extent of psychosocial burdens in this occupational group [2,3,4].

The nursing profession remains highly feminized—a structure rooted in the historical and social development of the field. This demographic pattern can influence work organization, division of duties, and how occupational stresses are experienced. At the same time, recent years have seen a gradual increase in the number of male nurses. Consequently, both biological sex and the gender-related social aspects of the profession may be relevant to interpreting research findings on the work environment and well-being of nursing staff [2].

In recent years, increasing attention has been devoted to the impact of the psychosocial work environment on the well-being of nurses working in ICUs. Evidence indicates that adverse psychosocial working conditions are associated with lower job satisfaction, a higher risk of burnout, and poorer psychological functioning among staff [2,3]. At the same time, it is emphasized that such burdens do not arise solely from the clinical nature of work; rather, they are substantially shaped by organizational factors, including the quality of the work environment, staffing adequacy, supervisory support, clarity of procedures, and the unit’s safety culture [4].

An important component of the psychosocial work environment in ICUs is staff exposure to workplace aggression and violence. Reviews of research in critical care show that ICU nurses experience both verbal and physical aggression from patients and their family members, alongside the underestimation and underreporting of violent incidents [5,6]. Exposure to workplace violence is linked to increased occupational stress, greater burnout symptoms, and reduced job satisfaction, thereby further exacerbating the negative psychosocial consequences of working in the ICU setting [7].

From a nursing management perspective, it is increasingly recognized that improving working conditions in ICUs requires actions that extend beyond individual stress-coping strategies. Systematic reviews suggest that organizational-level interventions—encompassing, inter alia, work organization, leadership support, communication, shift staffing, and the development of a safety culture—may yield more sustained improvements in staff well-being and stronger reductions in the adverse effects of psychosocial strain than approaches focused exclusively on the individual level [8].

The relationships between the psychosocial and organizational determinants of the work environment and the well-being of nursing staff can be explained through the Job Demands–Resources (JD-R) model and Conservation of Resources (COR) theory. The JD-R model posits that employees’ occupational functioning depends on the balance between job demands—such as time pressure, responsibility for critically ill patients, and high emotional load—and job resources, which include supervisory support, professional autonomy, quality of work organization, and a safety culture. A deficit in organizational resources paired with high job demands increases the risk of mental overload, burnout, and reduced job satisfaction. Similarly, COR theory indicates that employees strive to retain and protect their resources, whereas the actual loss or threat of loss of these resources leads to cumulative stress and impaired occupational functioning. In the intensive care unit environment, where exposure to numerous workplace stressors is particularly high, adequate organizational resources can buffer the negative effects of high demands and support both staff well-being and high-quality patient care [6,9,10,11].

In the present study, perceived occupational exposure was operationalized as the subjective evaluation of exposure to harmful, onerous, psychosocial, and organizational factors present in the work environment. This concept encompasses not only the objective presence of occupational hazards, but also how individual employees perceive them, which can subsequently influence their well-being, health behaviors, and job satisfaction [12].

Despite a growing body of research on the psychosocial work environment of nurses, most existing studies have examined single dimensions of occupational functioning—such as stress, burnout, or job satisfaction—without concurrently addressing perceived occupational exposure and organizational conditions [3,4,8]. Furthermore, available evidence suggests that the impact of organizational factors on staff well-being may be moderated by individual and organizational resources; however, findings remain somewhat inconsistent and vary depending on the organizational context and analytical models applied [7,10,11]. Very few studies among ICU nurses in Central and Eastern European countries have simultaneously examined both the psychosocial and organizational determinants of perceived occupational exposure and job satisfaction. The present study addresses this gap through a comprehensive assessment of these relationships using a standardized work-conditions assessment tool and logistic regression modeling.

The aim of the study was to evaluate the relationship between psychosocial and organizational work environment factors and the level of perceived occupational exposure and job satisfaction among intensive care unit nursing staff.

## 2. Materials and Methods

### 2.1. Research Design

The study employed a cross-sectional design and was conducted using a diagnostic survey method with an anonymous online questionnaire. The study design and the reporting of the results were developed in accordance with the STROBE guidelines (Strengthening the Reporting of Observational Studies in Epidemiology) for observational studies. The online format enabled voluntary participation and ensured the confidentiality of respondents’ answers.

### 2.2. Characteristics of the Study Group

The study included 226 nurses employed in intensive care units. Inclusion criteria were current employment in an ICU and practicing as a nurse. Exclusion criteria were incomplete completion of the questionnaire and lack of informed consent to participate in the study.

A purposive sampling method with elements of convenience sampling was used. Recruitment was conducted by distributing an electronic link to the survey among nursing staff employed in intensive care units and through online professional groups for anesthesiology and intensive care nurses.

Due to the exploratory nature of the study and the use of purposive sampling, no formal a priori sample size calculation was performed prior to the study. The response rate was also not calculated.

Sample size adequacy was evaluated during the statistical analysis phase, adhering to recommendations regarding the number of events per variable (EPV) for logistic regression models. In all analyzed models, the number of observations met the recommended EPV criterion, mitigating the risk of estimator instability and model overfitting.

### 2.3. Research Instruments

Two instruments were used: an author-developed questionnaire and the standardized Working Conditions questionnaire developed by the Central Institute for Labour Protection—National Research Institute (CIOP-PIB).

The author-developed questionnaire collected sociodemographic data (including age, sex, length of professional experience, and work system) and selected aspects of work organization in ICUs. The self-authored questions were single-item measures. Respondents evaluated: (1) subjective level of work-related stress, (2) overall job satisfaction, and (3) overall life satisfaction. Responses were provided on 5-point ordinal scales, ranging from very low to very high, or from strongly dissatisfied to strongly satisfied, depending on the question context. These items were supplementary and served to describe the study population, whereas the primary assessment of working conditions was conducted using the standardized CIOP-PIB questionnaire.

#### Standardized Instrument: Working Conditions Questionnaire (CIOP-PIB)

Working conditions were assessed using the standardized Working Conditions questionnaire developed by the Central Institute for Labour Protection—National Research Institute. This publicly available tool is intended for the subjective assessment of employees’ occupational exposure [13]. The questionnaire enables assessment across five domains of the work environment:

- exposure to harmful and burdensome factors,
- psychosocial factors,
- organizational factors,
- work-related health problems,
- opinions about work and life.

Sample questionnaire items included questions regarding time pressure and excessive workload, support from supervisors, recognition for a job well done, clarity of organizational procedures, and the opportunity to report occupational safety and health issues.

Subjective exposure to harmful and burdensome factors was assessed using a three-point scale (low, moderate, high), consistent with the occupational risk estimation scale applied in the PN-N-18002 standard. The remaining domains were evaluated using ordinal scales consistent with the original version of the questionnaire.

The questionnaire, together with a spreadsheet for coding and processing results, enables standardized data analysis in the areas of occupational exposure, psychosocial and organizational factors, and health problems. According to information obtained from the tool’s authors, the questionnaire is available for unrestricted use in scientific research. Based on CIOP-PIB results, aggregated variables were created and used in comparative and regression analyses. Based on the interpretive procedure specified for the questionnaire, aggregated variables were constructed to describe the level of psychosocial, organizational, and exposure to harmful or onerous factors. These were subsequently used in statistical analyses as dichotomous variables (high vs. low exposure).

Job satisfaction was assessed using a single item (“I am satisfied with my work”) from the “Opinions on Work and Life” section of the CIOP-PIB questionnaire. Responses were recorded on a 5-point scale (“yes”, “rather yes”, “rather no”, “no”, “don’t know”). For the purpose of logistic regression analysis, the variable was dichotomized as full job satisfaction (“yes”) versus all other responses.

For statistical analysis, aggregated variables were generated to represent exposure levels across the individual questionnaire domains (physical, psychosocial, and organizational factors), as well as health problems and opinions regarding work and life. These variables were created through a composite evaluation of responses within each respective domain and were utilized in further statistical modeling.

### 2.4. Study Procedure

Data were collected between January and May 2025 using the CAWI method (Computer-Assisted Web Interview), an online survey approach that facilitates rapid and efficient data collection. The questionnaire was distributed electronically, and the survey link was disseminated among ICU nurses and via social media platforms for anaesthesiology and intensive care nurses. Participation was voluntary and anonymous. Respondents were informed about the study aim and their right to withdraw at any stage.

Prior to completing the survey, all participants were informed about the study’s purpose and procedure, the voluntary nature of their participation, the option to withdraw at any stage, and confidentiality protocols. Commencing the survey was equivalent to providing informed consent to participate in the study.

To reduce potential systematic errors (bias), an anonymous survey format was employed, which may have lessened the influence of socially desirable responses. However, the voluntary nature of participation and the use of the CAWI method may have been associated with a risk of selection bias, resulting from the greater participation of individuals who were more engaged or more interested in the subject of the study. In addition, the use of self-report measurement tools entails the possibility of declarative bias.

### 2.5. Statistical Analysis

Statistical analysis was performed using IBM SPSS Statistics, Version 22.0 (IBM Corp., Armonk, NY, USA). Categorical variables were presented as frequencies and percentages, whereas continuous variables were described using means and standard deviations. Relationships between categorical variables were evaluated using Pearson’s chi-square test of independence, applying Yates’ continuity correction where appropriate.

The results of the association analysis were presented as frequencies and percentages. For the 2 × 2 analyses, odds ratios (OR) were calculated along with 95% confidence intervals (95% CI). The level of statistical significance was set at *p* < 0.05.

### 2.6. Ethical Considerations

The study was conducted in accordance with the Declaration of Helsinki and obtained approval from the Bioethics Committee (No. 24/2024). It constitutes a subgroup analysis of nurses within a broader project on stress and burnout among healthcare workers.

## 3. Results

### 3.1. Characteristics of the Study Group

The study included 226 intensive care unit (ICU) nursing staff. Women predominated (84.1%). The largest age groups were 41–50 years (37.2%) and 31–40 years (34.5%). More than 69% of respondents had over 10 years of professional experience. Most participants worked in a shift system (76.1%) and had completed a specialization in anaesthesiology and intensive care nursing (Table 1).

### 3.2. Psychosocial Factors of the Work Environment

Respondents reported a high level of psychosocial burden. The most frequently reported stressors were time pressure and excessive workload (94.7%), lack of understanding and support from supervisors (86.7%), and lack of recognition for work well done (81.4%). More than half of the participants frequently experienced persistent tension related to performing difficult and highly responsible tasks (Table 2).

High exposure to psychosocial risk factors was identified in 49.6% of respondents.

### 3.3. Organizational Factors of the Work Environment

Analysis of organizational factors showed that respondents most often rated access to medical care (78.8%) and managerial enforcement of occupational health and safety (OHS) rules (75.2%) positively. At the same time, a substantial proportion reported limited influence over the selection of protective measures and insufficient consideration of employees’ needs in workstation organization (Table 3).

High exposure to adverse organizational factors was identified in 69.9% of respondents.

### 3.4. Associations Between the Work Environment and Perceived Occupational Exposure

High perceived exposure to harmful and burdensome factors was reported by 25.7% of respondents. The analysis showed that sociodemographic variables such as age, length of professional experience, and work system did not significantly differentiate the level of perceived occupational exposure (*p* > 0.05). In contrast, a significantly higher level of exposure was observed among men compared with women (*p* < 0.001).

A significant association was also found between perceived exposure to adverse organizational factors and perceived occupational exposure (*p* = 0.002). Nurses working in a less favorable organizational environment more frequently reported high exposure to harmful and burdensome factors (Table 4).

The odds ratio (OR) for high organizational risk was 3.47 (95% CI: 1.54–7.81), indicating a more than threefold increase in the odds of high perceived occupational exposure compared to individuals working in a more favorable organizational environment.

## 4. Discussion

The most significant finding of this study was the demonstration of a high level of exposure to organizational and psychosocial factors among intensive care unit (ICU) nurses. A significant association was observed between perceived organizational risk and the level of occupational exposure. The obtained results suggest that the professional functioning of ICU nurses depends not only on exposure to traditional occupational hazards but also on work organization and psychosocial burdens. This indicates that improving occupational safety requires interventions aimed not only at mitigating physical hazards but also at optimizing work organization and enhancing psychosocial support for the staff.

The results of the present study highlight the significance of the organizational aspects of the work environment in relation to the perceived occupational exposure of ICU nursing staff. These findings align with the current body of research, which emphasizes the importance of organizational and managerial factors as key determinants of the professional functioning of nurses in critical care settings [1,2,3,4].

The analysis of the association between organizational risk and perceived occupational exposure revealed a significant relationship between these variables. Individuals characterized by high organizational risk had a more than threefold increase in the odds of experiencing high perceived occupational exposure compared to those with low organizational risk. International studies further indicate that the quality of the work environment, supervisory support, and the clarity of organizational procedures act as protective factors in intensive care units [5,6,7].

These findings can be interpreted within the framework of the Job Demands–Resources (JD-R) model, which posits that high job demands coupled with limited access to organizational resources lead to increased mental workload, reduced well-being, and lower job satisfaction. In the studied cohort of nurses, psychosocial and organizational factors were of paramount importance, as they may heighten the risk of occupational overload and adversely affect professional functioning. Concurrently, Conservation of Resources (COR) theory indicates that the prolonged depletion of resources—such as organizational support, perceived control, or opportunities for recovery—fosters cumulative stress and impairs employee performance [14]. The present results align with these theoretical assumptions and underscore the critical need for organizational interventions aimed at strengthening the resources available to nursing staff.

The high level of psychosocial burden among ICU nurses observed in this study is consistent with reports from other countries, where the ICU environment is repeatedly identified as one of the most psychologically demanding workplaces in healthcare. Evidence shows that ICU nurses more often than staff in other units report chronic stress, emotional overload, and symptoms of burnout, reflecting both the nature of caring for critically ill patients and the organizational conditions of work [7,8,15,16].

Exposure to workplace aggression and violence constitutes an additional—often underestimated—psychosocial burden characteristic of ICU settings. Although this study did not directly assess the frequency of violent incidents, the high levels of reported psychological tension and organizational strain may indirectly reflect the accumulation of such experiences. These findings are consistent with the literature indicating that exposure to aggression from patients and their relatives is associated with increased occupational stress, greater burnout, and reduced job satisfaction among critical care nursing staff. Future research should also consider evaluating other forms of negative interpersonal behaviors, such as mobbing, workplace incivility, and social exclusion, which may additionally impact the well-being and job satisfaction of nursing staff [17,18,19].

In the logistic regression model adjusted for selected sociodemographic variables, sex was statistically significant in relation to high perceived occupational exposure. This result should, however, be interpreted with considerable caution due to the small proportion of men in the sample and the wide confidence intervals of the estimates. The imbalance in group sizes reflects the demographic structure of the nursing profession but may have affected the stability of multivariable results. Available evidence suggests that differences in perceived occupational exposure may be more strongly related to variations in duties, task allocation, and more frequent involvement in physically or technically demanding procedures than to biological sex per se [20,21].

One key aspect of work organization that was not evaluated in the present study is shift length and total working time among nursing staff. Numerous studies indicate that extended shifts, particularly those lasting 12 h or longer, can increase fatigue, psychological burden, and the risk of errors, while reducing job satisfaction. Future research should incorporate a detailed assessment of working time organization—including shift length, weekly working hours, and overtime hours—to better elucidate their impact on perceived occupational exposure and the well-being of intensive care unit nurses.

Reports in the literature indicate that adverse organizational conditions, such as excessive workload, lack of recognition, limited influence on organizational decisions, and inadequate supervisory support, adversely affect the job satisfaction of ICU nurses [22,23,24]. An interesting observation is that high perceived occupational exposure was not a significant predictor of complete job satisfaction in the multivariable model. This finding may be interpreted in light of theories of job satisfaction in socially meaningful professions. The literature describes the phenomenon of maintaining relatively high job satisfaction despite significant burdens and risks, particularly among critical care nurses, where a sense of meaning in work, professional competence, and identification with the professional role may serve a buffering function [25,26].

A broader contextual factor to consider is the functioning of ICUs in the post–COVID-19 pandemic period. Studies indicate that the effects of prolonged organizational and emotional overload among ICU staff may persist after the pandemic, influencing risk perception, job satisfaction, and intentions to leave the profession. The present findings fit within this context and underscore the need for sustained system-level organizational interventions aimed at improving the ICU work environment [27,28,29].

From a nursing management perspective, the results highlight that interventions focused solely on reducing physical exposure may be insufficient if not accompanied by improvements in the psychosocial and organizational work environment. Actions involving leadership support, optimization of shift staffing, improved communication, and strengthening a culture of safety may play a key role in reducing perceived occupational exposure and maintaining job satisfaction among ICU nursing staff.

### Organizational Solutions to Improve the Health and Well-Being of ICU Nurses

The findings suggest that psychosocial and organizational workplace factors may play a crucial role in shaping perceived occupational exposure and job satisfaction among intensive care unit nurses. This implies that interventions focusing exclusively on individual-level coping strategies may be insufficient. To achieve sustained improvements in the health and well-being of ICU nursing staff, systemic organizational measures are essential.

First, optimizing workload should be prioritized. Ensuring adequate staffing standards, appropriate nurse-to-patient ratios, limiting overtime, and increasing the predictability of work schedules may significantly reduce time pressure and task overload—the stressors most frequently reported by respondents. International research shows that adequate staffing is associated with lower burnout, higher job satisfaction, and better patient-care outcomes [5,16]. However, it should be emphasized that the implementation of such solutions may be constrained by systemic factors, including staffing shortages, financial limitations, as well as existing organizational regulations and health policies. Consequently, initiatives aimed at improving the work environment should take into account both the capacity of individual healthcare facilities and the broader operational context of the healthcare system.

A second key area is management style and leadership quality. Supportive, participatory leadership models, regular feedback, recognition of staff achievements, and open communication promote perceived organizational justice, control over work, and psychosocial safety. Systematic reviews confirm that positive nursing leadership is an important predictor of job satisfaction and staff retention [21,24].

Another element should be the implementation of formal psychosocial support mechanisms. Regular post–critical incident team meetings (debriefing), peer support programs, and access to psychological consultations can reduce the accumulation of stress and lower the risk of burnout, particularly in critical care settings [8,28,30].

An important organizational innovation may also involve increasing nurses’ participation in decision-making processes. Shared governance models, participatory workplace design, and greater professional autonomy strengthen a sense of agency and meaning at work, translating into higher job satisfaction and lower intention to leave the profession [19,20,21].

Finally, remedial actions should be supported by systematic monitoring of the work environment. Regular assessment of psychosocial risks, organizational climate, and staff well-being indicators enables early identification of problems and planning of targeted interventions. Evidence syntheses indicate that organizational-level interventions are more effective and more sustainable than those focused solely on the individual [25,26,27].

The findings also hold significant practical implications. They indicate that interventions focused solely on mitigating physical hazards may be insufficient to enhance occupational safety among intensive care unit nurses. Equally essential is the reduction in psychosocial and organizational burdens through adequate staffing levels, rational work scheduling, stronger supervisory support, and the development of occupational stress prevention programs. Such measures may contribute not only to improving staff well-being, but also to enhancing the quality of patient care and reducing professional turnover.

The presented results should be interpreted in light of several limitations. First, the cross-sectional design precludes drawing causal inferences. Second, a purposive sampling approach with elements of convenience sampling was employed, and recruitment was partially conducted online, which may have increased the risk of selection bias—including self-selection bias—and limits the generalizability of the findings to the broader population of ICU nurses. Third, all data were gathered via self-report measures, carrying the risk of common method bias and subjective response variance. Additionally, the dichotomization of variables in the statistical analyses may have led to a loss of information and reduced statistical power.

Despite these limitations, this study provides valuable insights into the psychosocial and organizational determinants of perceived occupational exposure and job satisfaction among intensive care unit nurses. The findings serve as a foundation for planning future research utilizing prospective designs and larger, multicenter cohorts.

## 5. Conclusions

The present study indicates that psychosocial and organizational workplace factors may play a significant role in shaping perceived occupational exposure and job satisfaction among intensive care unit nurses. The findings suggest the need for further research into the effectiveness of organizational interventions aimed at improving the work environment and well-being of nursing staff. Prospective and multicenter studies evaluating causal relationships and the efficacy of implemented organizational solutions would be particularly valuable.

### Study Limitations

The study was cross-sectional in design, which precludes drawing causal inferences. A purposive sampling approach with elements of convenience sampling was employed, and recruitment was also conducted via social media, which may have increased the risk of selection bias—including self-selection bias—and limits the generalizability of the findings to the overall population of intensive care unit nurses.

All data were obtained using self-report methods, which carries the risk of common method bias and subjective response variance. Furthermore, the applied logistic regression analysis was exploratory in nature, and the use of dichotomized variables may have resulted in a partial loss of information and reduced statistical power.

Future research should incorporate larger, multicenter samples and utilize longitudinal designs as well as multidimensional models that account for a broader range of potential confounding factors.

## Figures and Tables

**Table 1:** Sociodemographic data of the surveyed group.

Variables		Frequency (n)	Percentage (%)
Age (years)	20–30	22	9.7
	31–40	78	34.5
	41–50	84	37.2
	Above 50	42	18.6
Gender	Female	190	84.1
	Male	36	15.9
Job seniority (years)	<5	40	17.7
	6–10	28	12.4
	11–20	78	34.5
	Above 20	80	35.4
Work shift system	Single-shift system (short shifts, 7 h 35 m)	40	17.7
	Single-shift system (12-h day shifts)	14	6.2
	Rotating shift system	172	76.1

**Table 2:** Psychosocial factors increasing psychological strain among ICU nursing staff.

Factor	Yes, Often (%)	Yes, Sometimes (%)	No (%)
Time pressure, excessive workload	79.6	15.0	5.3
Lack of understanding and support from supervisors	54.0	32.7	13.3
Lack of recognition for work well done	55.8	25.7	18.6
Persistent tension related to responsibility	59.3	20.4	20.4
Unclear instructions, poor work organization	31.0	39.8	29.2

**Table 3:** Selected organizational factors of the work environment.

Statement	Rather Yes/Yes (%)	Rather No/No (%)
Safety and health protection are as important as work efficiency	74.3	23.9
Management enforces OHS rules	75.2	19.5
Employees can report OHS problems without fear	69.0	25.6
Employees have influence over the choice of protective measures	42.5	47.8
Workstation organization takes employees’ needs into account	55.7	35.4

**Table 4:** Perceived exposure to organizational factors and perceived occupational exposure (n, %).

Exposure to Organizational Factors	High Occupational Exposure n (%)	Low Occupational Exposure n (%)	Total n (%)
High	50 (31.6)	108 (68.4)	158 (69.9)
Low	8 (11.8)	60 (88.2)	68 (30.1)
Total	58 (25.7)	168 (74.3)	226 (100)
*p* (χ^2^)	0.002		

## Data Availability

The data presented in this study are available on request from the corresponding author. The data are not publicly available due to privacy.
